# Treatment burden and regimen fatigue among patients with HIV and diabetes attending clinics of Tikur Anbessa specialized hospital

**DOI:** 10.1038/s41598-024-54609-5

**Published:** 2024-03-03

**Authors:** Oumer Sada Muhammed, Minimize Hassen, Melaku Taye, Eyob Beyene, Beshir Bedru, Melaku Tileku

**Affiliations:** 1 Department of Pharmacology and Clinical Pharmacy, College of Health Sciences, School of Pharmacy, Addis Ababa University, P.O. Box: 1176, Addis Ababa, Ethiopia; 2 Department of Internal Medicine, School of Medicine, College of Health Sciences, Addis Ababa University, Addis Ababa, Ethiopia; 3 Department of Clinical Pharmacy, School of Pharmacy, College of Medicine and Health Sciences, Wollo University, Dessie, Ethiopia

**Keywords:** Endocrinology, Health care

## Abstract

Nascent studies showed that patients with chronic medical illnesses such as diabetes mellitus (DM) and HIV/AIDS are highly vulnerable to face both treatment burden and regimen fatigue. However, an attempt made so far in this sphere in sub-Saharan African health care context is dearth. Thus, this study aimed to determine the level of treatment burden and regimen fatigue of diabetic and HIV patients attending adult diabetic and ART clinics of TASH and explore patients’ and health care workers’ propositions to reduce treatment burden and regimen fatigue. An explanatory sequential mixed methods study was conducted at the adult HIV and DM clinics of TASH, Addis Ababa, Ethiopia from February 01-March 30, 2022. Simple random and purposive sampling techniques were employed to select participants for quantitative and qualitative studies, respectively. Descriptive analysis was done to summarize the quantitative data. Logistic and linear regression analyses were performed to identify predictors of treatment burden and regimen fatigue, respectively. *P* value < 0.05 was considered statistically significant. Qualitative data was analyzed by using a thematic analysis. A total of 300 patients (200 diabetes and 100 HIV) were included in the quantitative study. For the qualitative study, 14 patients and 10 health care workers (six nurses and four medical doctors) were included. Participants' mean global Treatment Burden Questionnaire (TBQ) and Treatment Regimen Fatigue Scale (TRFS) score were 28.86 ± 22.13 and − 42.82 ± 17.45, respectively. Roughly, 12% of patients experienced a high treatment burden. The presence of two or more comorbidities (adjusted odds ratio [AOR] = 7.95, 95% confidence interval [CI] 1.59–39.08), daily ingestion of more than five types of prescribed medications (AOR = 6.81, 95%CI 1.59–29.14), and good knowledge about DM and/or HIV (AOR = 0.33, 95%CI 0.12–0.92) were predictors of treatment burden. Poor availability of medications (β = 0.951, *p* < 0.001) was the only predictor of regimen fatigue. Patients and health care workers primarily proposed to foster self-care efficacy, advance administrative services of the clinic and hospital, and improve healthcare system provision. The findings of this study unveiled that a considerable proportion of patients experienced low levels of treatment burden and regimen fatigue. This study showed that boosting the patients’ self-care efficacy, upgrading administrative services of the clinic and hospital, and promoting the healthcare system provision had enormous significance in reducing treatment burden and regimen fatigue. Therefore, when designing patient-specific healthcare interventions for both HIV and diabetic patients’ various factors affecting both treatment burden and regimen fatigue should be considered to achieve the desired goals of therapy.

## Introduction

Globally, chronic diseases like diabetes mellitus (DM) and HIV/AIDS are astoundingly increasing in almost epidemic proportions^1^. Unfortunately, adherence to lifelong complex treatment, continuous health care engagement, multiple lifestyle modifications, and adaptive coping skills are mandatory to adequately control these diseases^2^. In sub-Saharan Africa (SSA) countries including Ethiopia, these patients often face prodigious barriers such as out-of-pocket expenses, time spent traveling, attending clinical appointments, stigma, fear of disclosure, and drug stock-outs^2,3^. As a result, patients living with HIV and DM are highly vulnerable to facing treatment burden (TB) and regimen fatigue (TRF).

Treatment burden refers to the workload that a patient must manage to take care of their health and its impact on the patient’s daily life^4,5^. Treatment regimen fatigue refers to a decreased desire and motivation to maintain vigilance in adhering to a long-term treatment^6^. Both are novel clinical concepts that need to be well articulated in patients with DM and HIV/AIDS^3^. They can cause poor medication adherence which eventually leads to fatal clinical outcomes such as more hospitalizations, higher mortality, poorer health-related quality of life, worsening or recurrence of symptoms, and ineffective use of finite health resources^7,8^. Recent systematic review studies^9,10^ indicated that TB and TRF could be affected by numerous personal, disease, and treatment-related factors.

Although some qualitative studies^11–14^ in amalgamation with systematic reviews^4,6,7^ have been conducted to conceptualize TB and TRF in DM or HIV/AIDS, quantitative-based studies done on this sphere are dearth. Evidences indicated that TB and TRF had many common features, notwithstanding, they vary between specific countries and diseases as both are contextual concepts that depend on variety of factors such as personal characteristics like age gender, educational level, marital status, living condition, health literacy, and family support, illness duration or severity, treatment characteristics (e.g., medication type, number, and dose of medications), and financial cost of treatment^2,15^. So, direct extrapolation of findings from different Western countries is not a judicious option to apply in the SSA healthcare setup.

So far, quite a few studies have sought patients’ perspectives and propositions to ameliorate treatment onerousness and regimen fatigue. Nonetheless, almost all these studies were done in developed nations and thus could not be inferred to the SSA health care context. Hence, further exploration is required^2,16^. Moreover, the health care worker’s perspectives on these issues remain untapped. So, the current study was designed to fill these gaps of knowledge. Hence, this study aimed to determine the level of treatment burden and regimen fatigue of diabetic and HIV patients attending adult diabetic and ART clinics of TASH and explore patients’ and health care workers’ propositions to reduce treatment burden and regimen fatigue.

## Methods

### Study setting, study design and study period

This study used an explanatory sequential mixed methods design. Consequently, a quantitative (cross-sectional) investigation was followed by a qualitative study. From February 1 to March 30, 2022, the study was conducted at TASH's ambulatory ART and diabetes clinics in Addis Ababa, Ethiopia. TASH is Ethiopia's largest government-owned tertiary care, specialized, referral, and teaching hospital. It has 51 specialty and sub-specialty out-patient clinics that serve around 500,000 patients per year^17^. The ART and DM clinics are part of a larger network of specialized clinics that offer full care, including treatment and follow-up. The DM clinic is open three days a week (Monday, Wednesday, and Friday), but the ART clinic is open five days a week (excluding weekends). Every day, on average, 70 diabetics and 50 HIV patients come for follow-up.

### Study participants

All adult HIV and diabetes patients who visited TASH's respective ambulatory clinics on a regular basis and met the inclusion criteria during the study period were included. Patients were eligible for enrollment in this study if they were at least 18 years old, had been diagnosed with HIV or/and diabetes for at least 6 months prior to the study, had regular follow-up at TASH's ART and DM clinics, had been on treatment for at least 6 months, and could complete a written consent form. Patients with cognitive disabilities who may have difficulty understanding questions and critically sick patients who cannot tolerate interviews were excluded.

### Sample size calculation and sample size determination

The sample size was estimated using a single population proportion calculation with a 95% confidence level, 5% margin of error, 50% proportion of TB and TRF, expected number of source population (N = 4240), and 5% non-response rate. As a result, a total sample size of 370 patients was determined. 70 patients (30 from the diabetic clinic and 40 from the ART clinic) were excluded, leaving 300 patients (200 from the diabetes clinic and 100 from the HIV clinic) in the final analysis. The main reasons for exclusion were unwillingness to participate in the study and intolerance to complete in-depth interview.

To identify study participants who met the stated inclusion criteria for the quantitative investigation, a simple random selection procedure was used. As a sampling framework, the nurse appointment logbook was used. Patients were included in the research at random during their drug refill appointment. Purposive sampling was used to collect detailed information from patients and health care staff for the qualitative investigation. As a result, from each clinic, 7 patients and 5 health care staff were chosen. The qualitative study included 14 patients (7 females) and 10 health care providers (6 females and 4 males).

Six experienced nurses and four medical doctors (1 senior endocrine specialist, 1 senior infectious disease specialist, 2 fellowship residents, and 1 chief R3 resident) made up the health care team. Patients were chosen for in-depth interviews based on their TBQ and TRFS global scores, whereas health care personnel were chosen for key informant interviews based on their experience and knowledge with diabetes and HIV/AIDS management. After a brief explanation of the study by the matrons of the two clinics, eligible patients and health care personnel were reached through oral invitation and invitation letter, respectively. The participants who had only accepted the invitation offer were then contacted by phone to set up a convenient date and time for the interviews.

#### Study variables

Dependent variables: (1) treatment burden, (2) treatment regimen fatigue. Independent variables: (1) sociodemographic characteristics include age, sex, place of residence, occupation status, monthly income level, marital status, education level, smoking habit, and living conditions. (2) Clinical characteristics include duration of DM/HIV/AIDS, duration of treatment, severity of DM/HIV, presence and number of comorbidities, travel time, number of appointments, number of hospitalizations during the past 12 month, knowledge about DM/HIV/AIDS, and health literacy. (3) Treatment-related characteristics include total number of prescribed medications/pills, medications source, medication type, costs of medications, availability of medications, and ADR.

### Data collection instruments and procedure

The current investigation was divided into two basic parts. In phase I, standardized questionnaires were used to collect quantitative data on the patients' sociodemographic, clinical, and therapeutic features, as well as their level of TB and TRF. Patients' medical records were also checked to augment additional clinical data if needed. The Treatment Burden Questionnaire (TBQ-15) and Treatment Regimen Fatigue Scale (TRFS) were used to assess diabetic and HIV patients' treatment burden and regimen tiredness, respectively. The TBQ and TRFS were administered by two nurses having MSc degrees. On average, quantitative data gathering took 30 min.

The TBQ-15 is a well-validated universal psychometric instrument consisting of 15 items with scores ranging from 0 (no burden) to 10 (extremely high burden) that assesses the burden associated with medication management, self-monitoring, laboratory tests, doctor visits, the need for organization, administrative tasks, diet, physical activity, social impact, and financial burden of patients. It has five dimensions: medication-related hardship, administrative burden, financial burden, lifestyle modification burden, and social life-related burden^18^. The TRFS is a simple and reliable self-report psychometric instrument used to assess the amount of TRF in patients with chronic conditions who are on long-term treatment regimens. It consists of 22 items that examine three dimensions: treatment motivation, cynicism, and self-efficacy^19^.

Permission to utilize the TBQ and TRFS, as well as authorization to translate them, were secured from the original developers via Mapi Research Trust (MRT) and the corresponding author, who conducted the initial validation research, respectively. As previously described in our pioneering published work, a strict translation approach was employed^20^. In the current study, the Cronbach alpha values for the Amharic for Ethiopia versions of TBQ-15 and TRFS were 0.76 and 0.81, respectively. However, other than internal consistency, no additional psychometric testing was performed.

In phase II, semi-structured interviews were used to collect qualitative data on patients' and health care staff' suggestions for reducing TB and TRF. In-depth and key informant interviews lasting around 30 min were conducted utilizing an interview guide adapted from diverse literatures. Face-to-face cognitive interviews with 10 patients and 5 health care personnel were used to ensure the comprehensibility of the opening question and the usability of potential reformulations and explanations. Furthermore, the face and content validity of the data collection devices were investigated by four highly qualified professionals in clinical research and diabetes/HIV/AIDS management. Two clinical pharmacists with master's degrees and extensive prior experience in qualitative-based health research conducted the interviews face-to-face in a separate quiet room adjacent to the DM clinic's matron's head station.

Principal investigators adequately trained data collectors for two consecutive days on the strict application of study criteria, explanation of study objectives, obtaining written consents, implementation of sampling techniques, uniform interpretation of questions, and the confidentiality of collected data. To record information from the interviews, voice recorders and notebooks were employed. Interviews were performed until the information was exhausted. Finally, the results of both phases I and II were evaluated to provide a thorough understanding of TB and TRF.

### Data analysis

The quantitative data was input and cleaned in Epi Info version 4.6.0.2 before being exported and analyzed in the Statistical Package for the Social Sciences (SPSS) version 26. To summarize the categorical variables, frequencies and proportions were used. The mean, standard deviation, and/or median (IQR) of continuous variables were provided. Initially, normality tests utilizing plots and the Shapiro–Wilk test were used to choose relevant and robust statistical tests for continuous data. In addition, the variance inflation factor (VIF) was used to examine correlation among predictor variables. For excluding collinearity, a VIF of 10 was chosen as a cut point.

To analyze the relationship between treatment load and all independent variables and to identify candidates for multivariable analysis, binary logistic regression analysis was used. To find predictors of treatment burden, independent variables having *p* values less than 0.25 in univariable binary logistic regression analysis were re-entered into a multivariable binary logistic regression model. Statistical significance was defined as a *p* value of 0.05.

Univariable and multivariable linear regressions, on the other hand, were used to identify factors linked with regimen weariness. To begin, the statistical assumptions of linear regression analysis were put to the test. The Pearson R statistical test was used to calculate correlations between research variables. The multivariable linear regression analysis included all predictor variables with *p* values less than 0.25 in the univariable linear regression analysis. The overall model (F statistic, degrees of freedom, and *p*-value) was then reported, as was the variance explained by the overall model (adjusted r^2^) and any significant independent variables in the model (unstandardized beta coefficient, *p*-value).

Thematic analysis was used to assess the qualitative data. To aid with data analysis, the NVivo 12 qualitative data analysis program (QSR International Pty Ltd. Version 12, 2018) was employed. The data was first evaluated by verbatim transcribing the recorded data from Amharic to English, and then the transcript was classified and grouped into themes. Two investigators (OSM and MH) independently transcribed the audio recordings verbatim and read all of the participants' interview notes. The four investigators were in charge of coding, categorizing, and developing themes. Each theme's concepts were presented, narrated, and triangulated with quantitative findings.

### Operational definitions

Treatment Burden Global Score—the sum of all items scores of the questionnaire with ‘does not apply’ and missing answer considered the lowest possible score (0) [scores ranging from 0 to 150]. No Treatment Burden—a score of 0 for each item in the TBQ. Low Treatment Burden—a TBQ global score of < 59. High Treatment Burden—a TBQ global score of ≥ 59. Treatment Regimen Fatigue—waning commitment to continue with a prescribed long-term treatment. Limited Health Literacy—if participants ‘sometimes’ or ‘often’ or ‘always’ need reading help for written health materials related to DM and HIV. Poor Medications Availability —if medications are available ‘sometimes’, ‘rarely’, ‘not at all’ as self-reported by the patient. Poor Knowledge of DM/HIV—if patients responded ‘insufficient’ or ‘very insufficient’ when asked about their knowledge about HIV/AIDS and Diabetes (e.g., symptoms, disease progression) and its treatment protocols (e.g., possible side effects, expected benefits, other treatment options). Poor family support—if patients does not obtain family/caregiver support (physical, psychological, financial) in self-management of their disease. Comorbidity—presence of two or more concurrent illnesses other than the primary diseases (HIV-AIDS and Diabetes Mellitus).

### Ethical approval and informed consent

The study was approved by the Ethical Review Board (ERB) of Addis Ababa University, College of Health Sciences (25/03/2021; ERB No. 259/13/2021). The study protocol was performed in accordance with the Declaration of Helsinki. The aim and protocol of the current study were fully explained to all participants included in the study and written informed consent was obtained from all participants. All obtained data were treated confidentially.

## Results

### Sociodemographic characteristics of the study participants

A total of 300 patients (200 diabetes and 100 HIV) were included in the study. Out of the total, 162(54.0%) were female, 175(58.3%) were married, 115(38.3%) were unemployed, 253(84.3%) were from Addis Ababa, and 102(34.0%) had completed secondary school. The mean age of the participant was 49.76 ± 13.57 years (Table 1).

**Table 1:** Sociodemographic characteristics of HIV and diabetic patients attending adult ART and DM clinic of TASH, Addis Ababa, Ethiopia, February 01-March 30, 2022 (n = 300).

Variables	Category	Frequency	Percent
Age (years) [mean ± SD = 49.76 ± 13.57]	18–30	32	10.7
	31–60	203	67.7
	> 60	65	21.7
Gender	Male	138	46.0
	Female	162	54.0
Marital status	Single	61	20.3
	Married	175	58.3
	Divorced	28	9.3
	Widowed	36	12.0
Education level	No formal education	26	8.7
	Primary school completed	73	24.3
	Secondary school completed	102	34.0
	College and above	99	33.0
Employment status	Unemployed	115	38.3
	Government employed	83	27.7
	Private employed	16	5.3
	Self employed*	53	17.7
	Retired	33	11.0
Residence	Addis Ababa	253	84.3
	Outside Addis Ababa	47	15.7
Family support	No	80	26.7
	Yes	220	73.3
Living condition	Cohabiting*	235	78.3
	Alone	65	21.7
Smoking status	Never smoker	274	91.3
	Smoker (former/current)	26	8.7
Monthly income (in Eth Birr)	≤ 2000	146	48.7
	> 2000	154	51.3

Cohabiting*: living with a marriage partner, child or another partner like family and/or friends, Self-employed*: farmer, daily laborer, merchant, driver.

*Eth* Ethiopian, *SD* standard deviation.

### Clinical characteristics of the study participants

The median (IQR) duration of DM/HIV was 10 (6–16) years, and about 83.5% had type 2 DM. Most participants 212(70.7%) had to travel an hour and more to reach to the respective clinics. About 205(68.3%) of the participants were presented with other comorbid diseases (Table 2).

**Table 2:** Clinical characteristics of HIV and diabetic patients attending adult ART and DM clinic of TASH, Addis Ababa, Ethiopia, February 01-March 30, 2022 (n = 300).

Variables	Category	Frequency	Percent
Duration of DM/HIV [median (IQR) = 10(6–16)]	≤ 5 years	73	24.3
	6–10 years	84	28.0
	> 10 years	143	47.7
Type of DM* (n = 200)	Type 1	33	16.5
	Type 2	167	83.5
Health literacy (frequency of needing reading help related to DM/HIV)	Never	112	37.3
	Rarely	95	31.7
	Sometimes	48	16.0
	Often	27	9.0
	Always	18	6.0
Travel time to reach clinic [median (IQR) = 1.13(0.7–2.0)]	< 1 h	88	29.3
	≥ 1 h	212	70.7
No of Appo in the last 6 month [mean ± SD = 2.00 ± 1.45]	0–2	242	80.7
	≥ 3	58	19.3
Family Hx of DM (n = 200)	No	258	86.0
	Yes	42	14.0
Hx of hospitalization in the past 12 month	No	225	75.0
	Yes	75	25.0
Presence of comorbidity	No	95	31.7
	Yes	205	68.3
Treatment burden	Low (acceptable burden)	265	88.3
	High (unacceptable burden)	35	11.7

*DM* diabetes mellitus, *HIV* human immune deficiency virus, *No* number, *Hx* history, *Appo* appointment, *IQR* inter quartile range, *SD* standard deviation.

### Treatment related characteristics of the study participants

Participants took an average of 4.19 ± 3.19 pills daily. With regards to ADR, 69(23.0%) of them reported adverse drug effects. Nearly half of the study participants (52.3%) obtained their refill medications via health insurance system. Majority of the participants 138(46.0%) had two or more comorbid conditions. Out of which, half of them (50.0%) were presented with hypertension, then followed by cardiac diseases (39.0%), and dyslipidemia (13.7%) (Table 3).

**Table 3:** Treatment related characteristics of HIV and diabetic patients attending adult ART and DM clinic of TASH, Addis Ababa, Ethiopia, February 01-March 30, 2021 (n = 300).

Variables	Category	Frequency	Percent
Duration of DM/HIV treatment [median (IQR) = 10(5–15)]	≤ 5 years	82	27.3
	6–10 years	88	29.3
	> 10 years	130	43.3
Type of medication	Tablet/capsule only	180	60.0
	Injection only	32	10.7
	Tablet/capsule + injection	88	29.3
Source of medication	Free	83	27.7
	Payment	60	20.0
	Insurance	157	52.3
Monthly cost of medication	Less than 500 Birr	189	63.0
	500–1000 Birr	48	16.0
	Above 1000 Birr	63	21.0
Availability of medications	Always	116	38.7
	Often	69	23.0
	Sometimes	114	38.0
	Not at all	1	0.3
Presence of ADRs	No	231	77.0
	Yes	69	23.0
Knowledge about DM/HIV and its treatment protocols	Poor	69	23.0
	Good	231	77.0
No of prescribed medications [mean ± SD = 3.72 ± 2.21]	Up to 5 medications daily	218	72.7
	More than 5 medications daily	82	27.3
Daily no of pills [mean ± SD = 4.19 ± 3.19]	Up to 5 pills daily	194	64.7
	More than 5 pills daily	106	35.3
Total no of comorbidities [median (IQR) = 1(0–2)]	< 2	162	54.0
	≥ 2	138	46.0
Type of comorbidity	Hypertension	150	50.0
	Dyslipidemia	41	13.7
	Cardiac diseases	117	39.0
	Stroke	10	3.3
	Thyroid disorders	7	2.3
	Respiratory disorders	9	3.0
	Cancer	6	2.0
	Kidney failure	20	6.7
	Neurologic diseases	26	8.7
	Gastrointestinal diseases	8	2.7
	Musculoskeletal diseases	15	5.0
	Viral infections (HBV)	11	3.7
	Other diseases**	13	4.3

*ADRs* adverse drug reactions, *No* number, *HBV* Hepatitis B virus.

Other diseases**: schizophrenia, tuberculosis, erectile dysfunction, peripheral arterial disease, benign prostate hyperplasia, aortic aneurism.

### Description of treatment burden and treatment regimen fatigue

A mean global TBQ score of 28.86 (SD = 22.13) was reported by participants. Highest levels of treatment onerousness were reported in the administrative (mean = 9.05, SD = 9.42), medication (mean = 5.98, SD = 8.27) and social (mean = 5.93, SD = 6.10) domains. In contrast, lowest levels of treatment onerousness were displayed in the lifestyle change (mean = 4.06, SD = 5.05) and financial (mean = 3.84, SD = 4.22) domains (Table 4). About 265(88.3%) of the study participants exhibited low burden (TBQ score < 59), while only 35(11.7%) demonstrated high burden (TBQ score ≥ 59). With regard to regimen fatigue, participants’ self-reported mean global score of TRFS were − 42.82 (SD = 17.45).

**Table 4:** Analysis of five domains of TBQ among adult HIV and DM patients attending TASH.

Variables	Mean ± SD
1. Medication related burden	5.98 ± 8.27
Problems with the flavor, shape, or size of your drug, as well as discomforts induced by your injection (such as pain, bleeding, bruising, or scarring)?	1.61 ± 2.75
Problems caused by the number of times per day you must take your prescriptions (for example, once per day/twice per day)?	1.54 ± 2.88
Problems caused by the efforts required to remember to take your prescriptions (for example, not quitting taking medication when you are away from home, organizing and utilizing a pillbox……)?	1.48 ± 2.69
Problems caused by the precautions you must take when taking your prescriptions (for example, taking them at specified times or meals as suggested by your doctor, avoiding driving or lying down after taking meds, etc.)?	1.35 ± 2.41
2. Administrative related burden	9.05 ± 9.42
Problems with the time required to undergo lab tests and other associated procedures on a regular basis (for example, blood testing or radiology)?	2.89 ± 3.78
Problems with the time required to self-monitor your health state on a regular basis (for example, monitoring your blood pressure or BGL)?	0.71 ± 0.88
Problems with the time required to attend doctor visits and other medical appointments on a regular basis, as well as difficulty in locating health care professionals?	1.27 ± 2.58
Problems with your interaction with health care personnel during therapy (for example, not feeling listened to or having your opinions taken seriously)?	0.75 ± 2.05
Problems with scheduling medical appointments (doctor visits, laboratory testing, and other relevant tests) and time constraints for other life events?	1.09 ± 2.27
Problems with the administrative load of your healthcare system (for example, sorting out and filling out documents for hospitalization, reimbursements, and/or obtaining social services)?	2.33 ± 3.35
3. Financial Related Burden	3.84 ± 4.22
Problems with the financial load of your healthcare or treatment (for example, out-of-pocket payments or charges that are not covered by insurance)?	3.84 ± 4.22
4. Lifestyle change related burden	4.06 ± 5.05
Problems in making dietary modifications as prescribed by your doctor (for example, avoiding particular meals such as salty foods, reducing alcohol intake, quitting smoking…)?	2.36 ± 3.42
Problems with adhering to a doctor's physical exercise recommendations (for example, walking, jogging, swimming…)	1.69 ± 2.86
5. Social life related burden	5.93 ± 6.10
Problems with your treatment's impact on your social life (for example, asking support from family, friends, and other people in your everyday life, being ashamed to take your prescription in public…)?	2.18 ± 3.32
“The need for regular medical healthcare reminds my health problems.”	3.75 ± 4.04

### Factors associated with treatment burden

The multivariable binary logistic regression model unveiled that presence of two and more comorbidities adjusted odd ratio [AOR] = 7.95, 95% CI 1.59–39.08), daily ingestion of more than five types of prescribed medications (AOR = 6.81, 95% CI 1.59–29.14), and good knowledge about DM/HIV and their treatment protocols (AOR = 0.33, 95% CI 0.12–0.92) were found to be predictors of higher burden of treatment (Table 5).

**Table 5:** Univariable and multivariable logistic regression analysis of factors associated with treatment burden among HIV and diabetic patients attending ART and DM clinic of TASH, Addis Ababa, Ethiopia, February 01-March 30, 2022 (n = 300).

Variables	Treatment burden		COR (95% CI)	AOR (95%)	*P* value
	Low, n (%)	High, n (%)			
Education					
No formal education	22 (84.6)	4 (15.4)	1	1	1
Primary school	69 (94.5)	4 (5.5)	0.32 (0.07–1.38)	0.49 (0.08–3.02)	0.445
Secondary school	89 (87.3)	13 (12.7)	0.80 (0.24–2.71)	2.43 (0.49–11.93)	0.273
College and above	85 (85.9)	14 (14.1)	0.91 (0.27–3.03)	2.05 (0.37–11.38)	0.411
Marital status					
Married	157 (89.7)	18 (10.3)	1	1	1
Single	55 (90.2)	6 (9.8)	0.95 (0.36–2.52)	1.31 (0.35–4.96)	0.688
Divorced	25 (89.3)	3 (10.7)	1.05 (0.29–3.81)	1.67 (0.30–9.13)	0.556
Widowed	28 (77.8)	8 (22.2)	2.49 (0.99–6.28)	1.79 (0.49–6.53)	0.374
Knowledge of Dx					
No	56 (80.0)	14 (20.0)	1	1	1
Yes	209 (90.9)	21 (9.1)	0.41 (0.19–0.84)	0.33 (0.12–0.92)	0.034*
Time to reach clinic					
< 1 h	83 (94.3)	5 (5.7)	1	1	1
≥ 1 h	182 (85.8)	30 (14.2)	2.74 (1.03–7.31)	1.76 (0.54–5.77)	0.353
No of comorbidities					
< 2	159 (98.1)	3 (1.9)	1	1	1
≥ 2	106 (76.8)	32 (23.2)	16.00 (4.78–53.59)	7.95 (1.59–39.08)	0.012*
Hypertension					
No	146 (97.3)	4 (2.7)	1	1	1
Yes	119 (79.3)	31 (20.7)	9.51 (3.27–27.69)	3.32 (0.93–11.90)	0.065
Cardiac diseases					
No	173 (94.5)	10 (5.5)	1	1	1
Yes	92 (78.6)	25 (21.4)	4.70 (2.16–10.21)	0.93 (0.27–3.17)	0.900
ADR					
No	208 (90.0)	23 (10.0)	1	1	1
Yes	57 (82.6)	12 (17.4)	1.91 (0.89–4.06)	1.37 (0.51–3.72)	0.532
No of appo in 6 mon					
0–2	218 (90.1)	24 (9.9)	1	1	1
≥ 3	47 (81.0)	11 (19.0)	2.13 (0.97–4.64)	0.91 (0.29–2.83)	0.868
Total prescribed med					
≤ 5 Medications	205 (94.0)	13 (6.0)	1	1	1
> 5 Medications	60 (73.2)	22 (26.8)	5.78 (2.75–12.16)	6.81 (1.59–29.14)	0.010*
Total no of pills/day					
≤ 5 Pills	180 (92.8)	14 (7.2)	1	1	1
> 5 Pills	85 (80.2)	21 (19.8)	3.18 (1.54–6.55)	0.61 (0.15–2.41)	0.480
Medication cost					
< 500 Birr/month	174 (92.1)	15 (7.9)	1	1	1
500–1000 Birr/month	42 (87.5)	6 (12.5)	1.66 (0.61–4.53)	0.75 (0.20–2.77)	0.666
> 1000 Birr/month	49 (77.8)	14 (22.2)	3.31 (1.49–7.33)	1.17 (0.30–4.51)	0.821
Disease duration					
≤ 5 years	61 (83.6)	12 (16.4)	1	1	1
6–10 years	73 (86.9)	11 (13.1)	0.77 (0.32–1.86)	1.49 (0.47–4.76)	0.497
> 10 years	131 (91.6)	12 (8.4)	0.47 (0.19–1.09)	1.27 (0.40–3.95)	0.686
Drug availability					
Good	170 (91.9)	15 (8.1)	1	1	1
Poor	95 (82.6)	20 (17.4)		1.64 (0.58–4.69)	0.354
Medication source					
Payment	49 (81.7)	11 (18.3)	1	1	1
Insurance	139 (88.5)	18 (11.5)	0.58 (0.26–1.31)	0.34 (0.09–1.30)	0.599
Free	77 (92.8)	6 (7.2)	0.35 (0.12–0.99)	1.61 (0.28–9.38)	0.116

*COR* crude odds ratio, *AOR* adjusted odds ratio, *CI* confidence interval, *Appo* appointment, *Dx* disease, *ADR* adverse drug reaction, *No* number, *mon* month, *Med* medication.

*Significant at p < 0.05.

### Factors associated with treatment regimen fatigue

Correlational analysis examining the association between potential antecedent factors and TRF indicated that number of comorbidities (r = 0.125; *p* = 0.030) was significantly correlated with TRF (see supplementary Table S1). The multivariable analysis demonstrated that eight percent of the variation in overall TRF was explained by the set of independent variables (adjusted R^2^ = 0.081, F (16, 283) = 2.655, *p* < 0.001). The only variable that made significant contribution (β = 0.951, 95% CI [0.49, 1.42], *p* < 0.001) to the prediction of TRF was poor medication availability (Table 6).

**Table 6:** Univariable and multivariable linear regression analysis of predictors of regimen fatigue among HIV and diabetic patients attending ART and DM clinic of TASH, Addis Ababa, Ethiopia, February 01 to March 30, 2022 (n = 300).

Variables	Univariable analysis	*P* value	Multivariable analysis	*P* value
	Beta coefficient (95% CI)		Beta coefficient (95% CI)	
No of comorbidity	0.175 (0.02, 0.03)	0.030	0.046 (− 0.19, 0.28)	0.703
No of appointment	0.106 (− 0.04, 0.25)	0.145	0.013 (− 0.14, 0.17)	0.870
Travel time	0.038 (− 0.01, 0.09)	0.120	0.005 (− 0.05, 0.06)	0.861
Total no of med	0.064 (− 0.03, 0.16)	0.177	− 0.052 (− 0.18, 0.08)	0.426
Sex				
Male	1	1	1	1
Female	0.280 (− 0.13, 0.69)	0.184	0.054 (− 0.38, 0.48)	0.804
Residence				
Addis Ababa	1	1	1	1
Outside AA	0.491(− 0.08, 1.06)	0.089	0.219 (− 0.44, 0.88)	0.514
Education				
No formal edu	1	1	1	1
Formal edu	− 0.869 (− 1.59, − 0.14)	0.020	− 0.505 (− 1.27, 0.26)	0.195
Occupation				
Unemployed	1	1	1	1
Employed	− 0.312 (− 0.72, 0.10)	0.138	− 0.198 (− 0.62, 0.23)	0.359
Hypertension				
No	1	1	1	1
Yes	0.397 (− 0.01, 0.81)	0.058	− 0.006 (− 0.54, 0.53)	0.982
Knowledge of Dx				
Good	1	1	1	1
Poor	− 0.733 (− 1.22, − 0.25)	0.003	− 0.459 (0.96, 0.04)	0.071
ADRs				
No	1	1	1	1
Yes	0.444 (− 0.05, 0.93)	0.075	0.345 (− 0.15, 0.84)	0.170
Hospitalization				
No	1	1	1	1
Yes	0.417 (− 0.06, 0.89)	0.085	0.159 (− 0.33, 0.65)	0.522
Med availability				
Good	1	1	1	1
Poor	1.082 (0.68, 1.49)	< 0.001	0.951 (0.49–1.42)	< 0.001*
Source of med				
Free/insurance	1	1	1	1
Payment	0.418 (− 0.09, 0.93)	0.111	0.287 (− 0.32, 0.89)	0.349
Type of med				
Tablet/injection	1	1	1	1
Tablet + injection	0.328 (− 0.12, 0.78)	0.154	0.029 (− 0.46, 0.52)	0.908
Cost of med				
< 500 Birr/month	1	1	1	1
≥ 500 Birr/month	0.526 (0.10, 0.95)	0.015	− 0.045 (− 0.59, 0.50)	0.873

*AA* Addis Ababa, *No* number, *ADR* adverse drug reaction, *Edu* education, *Med* medication, *Dx* disease, hospitalization.

*Significant at p < 0.05, beta coefficient: unstandardized beta coefficient (β).

### Qualitative analysis of patients’ and health care workers’ propositions to decrease treatment burden and regimen fatigue

Three major themes were extracted from patients and health care workers propositions on how burden of treatment and regimen fatigue could be ameliorated. (1) Fostering self-care efficacy, (2) Advancing the administrative services of the clinic and the hospital, and (3) Improving the healthcare system provision. Then, subthemes along with excerpts emerged in each key theme (see Supplementary Tables S4 and S5).

#### Theme 1: fostering self care efficacy

Patients proposed to modify the complexity of treatment regimens and contents of counseling tips as a means of developing self-care efficacy. To this end, one of the participants underscored that:

*In the process of patient consultation for HIV patients, awareness campaigns should be incorporated with special emphasis on the topics of stigma and fear of disclosure (P-11).*

Consonant to the patient’s perspective, health care workers advocate improving consultation content, providing patient education, establishing formalized patient support group system, and availing fixed-dose combination (FDC) medications to improve self-care management.

Regarding role of patient education, an experienced infectious disease fellow specialist serving in the HIV clinic iterated that:

*I think counseling on prescribed medication adherence along with its potential merits outweighs mere prescribing (HCP-2).*

#### Theme 2: advancing the administrative services of the clinic and the hospital

Major issues emphasized by participants to upgrade the administrative services of the clinic or hospital include improving waiting area of the clinics, enhancing medication availability, reducing patient flow, promoting infrequent changing of physicians, and availing functional laboratory tests.

Most patients repeatedly iterated the importance of availing fully functional laboratory tests and hiring qualified laboratory technicians. To this end, one of the participants asserted that:

*Surprisingly, crucial laboratory tests like HgbA1c, thyroid function tests, and viral load are unavailable in this big referral hospital causing us to look forward to other high-priced private institutions capable of undergoing these tests (P-12).*

In conformity to the above statement, another participant stated that:

*The queue for undergoing lab tests is prodigious but the number of working lab technicians are few (usually not more than two). Owing to this, the service provided is quite slow. To overcome this, competent lab technicians should be hired (P-2).*

In conformity to the patients’ proposition, health care workers proposed to extend follow-up schedule and improve laboratory services of the hospital. To this end, an experienced endocrine fellow specialist serving in the diabetic clinic underscored as follows:

*I heard frequent complaints from patients pertaining to the service provided in the hospital’s laboratory. To surmount this, many competent and well qualified lab technicians should be employed. If possible, because the diabetic clinic is one of the heavily burdened clinics in TASH, it is good to establish a separate lab for the clinic where only DM related tests will be performed (HCP-1).*

#### Theme 3: improving the health care system provision

Patients conceived that the ripest way for boosting health care system provision is via obtaining social support, developing communication skills of non-medical staffs, and strengthening health insurance system. Regarding social support, one participant claimed that:

*I believe that social help from a government and/or sponsoring organization in the form of financial aid or job opportunity is a weapon for transforming the health care system (P-3).*

As reiterated by the health care workers, establishing a link between health insurance office and Kenema pharmacy, advancing the electronic record (I-Care) system, launching a well-organized health insurance system, and providing financial support have paramount role to upgrade the provision of the health care system.

With respect to the I-care system, one of the nurses who had 10-year work experience in the diabetic clinic asserted that:

*The updated I-Care software prescription has no empty space for writing patients’ medical diagnosis and putting prescriber signature. As a result of this, dispensing pharmacists often considered it as incomplete prescription and return patients to respective physician for rectification (HCP-2).*

## Discussion

This study is the pioneer to explore the treatment burden and regimen fatigue of patients with HIV/AIDS and DM from the perspective of both health care workers and patients in SSA health care context, particularly in Ethiopia, using a mixed method study approach. Unfortunately, quite a few quantitative based-studies are available to compare our results owing to the novelty of conceptualizing and measuring TB and TRF in the aforementioned disease contexts.

The current study finding revealed a mean global TBQ of 28.86 (SD = 22.13). This finding is consonant with the findings of a study conducted in Switzerland (mean = 26.8, SD = 18.6)^21^. Though the specific disease context is different (heart failure), the finding is consistent with a recent study done in Ethiopia using similar patient-reported outcome measure [TBQ] (mean = 27.2, SD = 19.4)^20^. Conversely, it is smaller as compared to the studies conducted in USA, Cleveland State, (mean = 37.0, SD = 24.5)^22^, Australia (mean = 56.5, SD = 34.5)^23^, Qatar (Median = 40.5, IQR = 38)^24^, Côte d’Ivoire (mean = 33.3, SD = 19.6)^2^, and larger than a study done in USA, Ohio State, (mean = 22.8, SD = 24.6)^25^. The incongruity might be ascribed to the differences in the quality of health care provided, fragmented, and disorganized health care system provision, and varied economic capability. Our study determined the level of TB/TRF solely from the perspective of HIV and diabetic population alone.

In this study, highest level of TB was reported in administrative (mean = 9.05, SD = 9.4), medication (mean = 5.98, SD = 8.2) and social (mean = 5.93, SD = 6.1) domains, while lowest value of TB was displayed by lifestyle change (mean = 4.06, SD = 5.0) and financial (mean = 3.84, SD = 4.2) domains. This finding is incongruent with studies done in Australia^23^ that reported highest TB on financial, lifestyle, social, administrative, and medication domains, respectively and in Qatar^24^ that showed highest TB on medication, lifestyle, administrative, social and financial domain, respectively and in Ethiopia^20^ that showed administrative, financial, lifestyle, social, and medication domains, respectively. The possible reason behind such discrepancies could be attributed to the differences in the quality of the health care provided, disorganized healthcare system, economic factors, and specific disease context.

The present study findings revealed that about 265(88.3%) of participants reported low burden, and only 35(11.7%) indicated high burden. This finding is incongruent to Tran’s et al*.*^26^ study, where roughly 47% experienced low, 28% moderate, and 24% high onerousness. Contrary to our finding, Bekalu’ et al*.*^27^ recently found moderate (58.9%) to high (26.2%) level of medication related burden and Baah-Nyarkoh et al*.*^28^ indicated 69.3% patients as having minimal burden in diabetic patients. The inconsistency could be attributed to variations in the characteristics of the study subjects. For instance, Tran's and Bekalu’s incorporated a large sample comprised of slightly older, and highly educated subjects. The discrepancy could also be colligated to difference on the cut-off value used to label patients as low and high burden. Unlike most previous studies that reckoned TBQ as a continuous variable, ours considered it as dichotomous categorical variable [low vs High] by referring the current scoring interpretation developed by the original developers. Nonetheless, this finding is in keeping with Pedersen et al.^29^ and Hassen et al.^20^ studies that revealed 13% and 12% patients exhibited high treatment burden.

With regard to TRF, the finding of this study reported a mean global TRFS of − 42.82 (SD = 17.45). This finding is in conformity with Claborne et al*.* that reported an overall low level of TRF (mean =  − 41.28, SD = 21.08)^19^. Claborn et al.’ s mean level of TRF was only 2 points greater than the mean observed from the current study.

According to the finding of this study, patients who presented with two and more concurrent illnesses have higher TB compared with their counterparts. This finding is consonant with previous studies by Sav et al.^23^, Morris et al.^30^, Al-mansouri et al.^24^, and Schreiner et al.^25^ which unveiled that as the number of concurrent illnesses increases the level of treatment onerousness increases proportionally. This is possibly due to the need of polypharmacy, complex treatment regimens, erratic medication taking behavior, and uninterrupted motivation. This study revealed that more intake of prescribed medications (> 5 types) was strongly linked with higher TB. Similar observations were noted in studies by Morris et al., and Hassen et al., which showed that high TB was strongly associated with more prescribed regular medications^20,30^.

Furthermore, this study unveiled that having sufficient knowledge about DM/HIV and its treatment protocol and availability of medications was strongly associated with lower TB. This finding in agreement with Hassen et al., study^20^ though the specific disease context is different (DM/HIV vs Heart Failure). Even with differences in disease context, knowledge and medication availability have massive impact for both diseases owing to their chronic nature. Moreover, this finding is consistent with previous qualitative studies^31,32^.

Regarding TRF, the finding of this study demonstrated that poor availability of medications was significantly associated with TRF of HIV and diabetic patients. This finding is novel because so far, the available studies did not quantitatively examine its association with TRF. However, this finding was supported by previous qualitative studies by Claborne et al.^33^, and Crowford et al.^34^, which showed that TRF may be precipitated by the agglomerative effects of treatment, physical, psychosocial, cognitive, and economical-related factors.

As evidenced from the qualitative finding of this study, patients genuinely proposed for boosting self-care efficacy, administrative services of the clinic and/or the hospital, and health care system provision to alleviate and/or prevent the occurrence of TB and TRF. This finding is in line with a previous study by Ting et al., which indicated that patients suggestions to ameliorate treatment onerousness were focused on better provision of health care services and access, and rendering a less burdensome regimen^32^. Perhaps, this finding is supported by a study by Tran et al. which unveiled that patient proposed to improve personal care and the hospital’s organization and the health care system^2^. Apparently, the present study found that health care workers perspectives on decreasing TB and TRF strongly aligns with patients’ perspectives, especially concerning to the laboratory set-up and service.

Unfortunately, some of the propositions raised by patients in the current study were comparable as those underscored by patients residing in developed countries though clear difference was noted in terms of the magnitude of their impact on patients’ lives. For example, financial constraints associated with transportation and refilling medications was unbearable in this study and thus most patients emphasized having social and/or financial support and availing of less costly medications. In contrary, in developed countries like France and Chile, health care services are delivered free of charge to ambulatory patients presenting with any chronic diseases. Indeed, the variety of patients and health care workers propositions spotlighted that there is no ‘one-size-fits-all’ approach to minify TB and TRF and healthcare must be tailored to fit each patient’s context.

Finally, this study has some limitations that need to be acknowledged. First, it investigated TB and TRF at a single point in time cross-sectionally. Second, some variables such as smoking habit, disease duration, daily number of pills were obtained directly from patients which may cause social desirability and recall bias. Third, interviewer administration approach was applied to collect the quantitative data using TBQ and TRFS that may eventually lead to social desirability bias. To trim this risk, all data collectors strictly followed the interview protocols and avoided any personal knowledge, beliefs, and influences. Fourth, it was confined to HIV and diabetic patients who could speak Amharic language only and thus may not be generalized to other cultures or countries with other types of chronic medical illnesses. Fifth, the TBQ-15 and TRFS tools were culturally adapted and implemented, however, except internal consistency no further psychometric testing was done.

## Conclusion

The findings of this study unveiled that a considerable proportion of patients faced low levels of TB and TRF. Our finding revealed that the presence of two or more comorbidities, daily intake of more than five types of prescribed medications, and disease knowledge were found to have statistically significant associations with high TB. This study also showed that poor medication availability was significantly associated with TRF. The qualitative findings of this study unveiled that fostering self-care efficacy, administrative services of the clinic/hospital, and the health care provision had paramount importance in reducing TB and TRF. Multiple factors that increase TB and TRF should be considered when designing specific healthcare interventions toward HV and diabetic patients to achieve the desired goals of therapy.

### Supplementary Information

Supplementary Information 1.

Supplementary Tables.

## Data Availability

The datasets used and/or analyzed during the current study are available from the corresponding author upon reasonable request.

## Abbreviations

AIDS: Acquired immune deficiency syndrome
ART: Antiretroviral therapy
DM: Diabetes mellitus
FDC: Fixed dose combination
HIV: Human immune deficiency virus
MRT: Mapi research trust
SPSS: Statistical package for social scientists
TASH: Tikur Anbessa specialized hospital
TBQ: Treatment burden questionnaire
TRFS: Treatment regimen fatigue scale
VIF: Variance inflation factor
