# Supplementary material for: Treatment burden and regimen fatigue among patients with HIV and diabetes attending clinics of Tikur Anbessa specialized hospital
Source: Sci Rep. 2024 Mar 3;14:5221. doi: 10.1038/s41598-024-54609-5 (PMC10909857; doi:10.1038/s41598-024-54609-5)
Supplement: Supplementary file 2 — Supplementary Tables. [file 41598_2024_54609_MOESM2_ESM.pdf]

Treatment Burden and Regimen Fatigue Among Patients with HIV and Diabetes Attending ART and Diabetic Clinics of Tikur Anbessa Specialized Hospital: An Explanatory Sequential Mixed-Methods Study

Oumer Sada Muhammed[1], Minimize Hassen[2], Melaku Taye[3], Eyob Beyene[4], Beshir Bedru[5], Melaku Tileku[6]*

Supplementary Table S1. Correlation between continuous predictor variables and TRF

**Variable**	1	2	3	4	5	6	7	8
1. TRF	-							
2. Age	-0.008	-	0.372**				0.433**	0.433*
3. No of Comorbid	0.125*		-				0.640**	0.619**
4. Duration of Dx	-0.052		-0.260**	-	-0.272**		-0.278**	-0.297**
5. No of App in 6m	0.084		0.225*		-		0.326**	0.304**
6. Travel Time	0.090					-		
7. Total Medication	0.078						-	0.870**
8. No of pills/day	0.073							-

**=Correlation is significant at the 0.05 level; **=Correlation is significant at the 0.01 level; No: Number; Dx: Disease; App: Appointment; sqrt: square root; TRF: Treatment Regimen Fatigue*

Supplementary Table S2: Model Goodness of Fit Test Using Chi-Square

Test Statistics	
	TRFSQSUM_Sq
Chi-Square	233.613[a]
df	61
Asymp. Sig.	.000
a. 62 cells (100.0%) have expected frequencies less than 5. The minimum expected cell frequency is 4.8.	

Supplementary Table S3: Linear Regression Output After Performing Interaction Test

Coefficients[a]								
Model		*Unstandardized Coefficients*		*Standardized Coefficients*			*95.0% Confidence Interval for B*	
		B	*Std. Error*	*Beta*	*t*	*Sig.*	*Lower Bound*	*Upper Bound*
1	(Constant)	5.014	.463		10.831	.000	4.103	5.925
	Sex	.053	.218	.015	.244	.807	-.376	.483
	Residence	.242	.337	.048	.716	.474	-.422	.905
	Education_N	-.527	.391	-.082	-1.350	.178	-1.297	.242
	Occupation_N	-.197	.216	-.054	-.913	.362	-.623	.228
	HTN	-.004	.272	-.001	-.015	.988	-.539	.530
	KnowCondTT	-.451	.253	-.105	-1.780	.076	-.949	.048
	ADRs	.349	.251	.081	1.391	.165	-.145	.842
	Availablity_Cat	.947	.236	.254	4.009	.000	.482	1.413
	SourceMed_N	.309	.309	.068	1.002	.317	-.298	.917
	CostofMed_N	-.058	.279	-.016	-.209	.834	-.608	.491
	TypeofMed_N	.019	.249	.005	.077	.938	-.471	.509
	HxofHospital	.159	.248	.038	.642	.522	-.329	.648
	NoComorbidty_C	.043	.121	.030	.352	.725	-.195	.281
	NumberAppo_C	.009	.078	.007	.111	.911	-.144	.161
	TravelTime_C	.005	.029	.012	.182	.856	-.051	.061
	NoPrescMed_C	-.051	.066	-.063	-.780	.436	-.181	.078
	Interaction_New	-.006	.009	-.036	-.640	.523	-.023	.012
a. Dependent Variable: TRFSQSUM_Sq								

Supplementary Table S4. Patients' Propositions to Decrease Treatment Burden and Regimen Fatigue

**Theme-1: Fostering self-care efficacy**	
**Subtheme-1: Treatment regimen modifications**	
P-1	*I am tired of taking so many pills daily. I prefer changing regimens with smaller pills, pills with a better taste, and medications requiring a less strict dosage schedule.*
P-2	*My motivation to take my medication has decreased over time due to unpalatable tests from my medication and intolerable side effects. It is better to prescribe drugs with palatable dosage forms and minimal side effects.*
**Subtheme-2: Patient counseling tips modification**	
P-3	*Physicians should provide more information about the care I am receiving, mode of transmission, sign and symptoms of my condition along with the treatment protocols.*
P-11	*In the process of patient consultation for HIV patients, awareness campaigns should be incorporated with special emphasis on the topics of stigma and fear of disclosure.*
**Theme-2: Advancing the administrative services of the clinic and/or hospital (TASH)**	
**Subtheme-1: Improving waiting area of the clinic**	
P-4	*The waiting area of the clinic and the pharmacy should be comfortable for patients who have concomitant disease, and who could not resist large queues for a longer period.*
P-2	*The waiting area of the ART clinic is too suffocated and has no adequate sits.*
**Subtheme-2: Enhancing medication availability**	
P-1	*Only cheap medications are available at the diabetic pharmacy and OPD pharmacy. As a result, I am obliged to obtain my medications from private community pharmacies.*
P-9	*The approach of the dispensing pharmacists is often annoying even if the medications are available in the pharmacy store by any chance.*
P-5	*Recently, dispensing pharmacists denied patients with health insurance from accessing brand medications. In fact, this is irritating to me.*
**Subtheme-3: Reducing patient flow**	
P-6	*To accommodate the high patient flow in the diabetic clinic, it is good to change the follow-up schedule from half day to a full day.*

P-9	*It is better to increase the number of physicians in each follow-up schedule to make it proportional with the incoming patient flow.*
**Subtheme-4: Improving frequent changing of physicians during each follow-up visit**	
P-1	*I believe that having a permanent doctor during each follow-up visit would enable the clinician to consistently assess the successive physical and clinical progress of patients.*
P-10	*Despite of having healthy relationship with every physician I encountered, I do not get the same physician in my consecutive follow-up visits.*
**Subtheme-5: Availing fully functional laboratory tests in the hospital**	
P-12	*Surprisingly, crucial laboratory tests like HgbA1c, thyroid function tests, and viral load are unavailable in this big referral hospital causing us to look forward to other high priced private institutions capable of undergoing these tests. To the best of possible, these tests should be available in the hospital and be fully functional.*
P-2	*The queue for undergoing laboratory tests is prodigious but the number of working laboratory technicians are very few (usually not more than two). Owing to this, the service provided is quite slow. To overcome this, competent lab technicians should be hired taking in to account the actual patient flow.*
**Theme-3: Improving the health care system provision**	
**Subtheme-1: Obtaining social support**	
P-3	*I believe that social help from the government and/or sponsoring organization in the form of financial aid or job opportunity is a preventive weapon for transforming the health care system.*
P-13	*When all prescribed medications aren't available in a single pharmacy, collecting from different community pharmacies is tiresome due to transportation problem and cost. In such cases, free transportation service should be arranged by the hospital especially for financially limited patients.*
**Subtheme-2: Improving the communication skills of non-medical staffs**	
P-6	*The behavior of the security guards assigned in the waiting room of the clinic should be strictly followed by the respective concerned body because they make the environment uncomfortable due to their poor communication skills with patients.*

P-9	*Some people get ambulatory health care service via social advantages while others are waiting for their queue. In fact, this is really weird and it requires thorough supervision from the responsible organ.*
**Subtheme-3: Strengthening and organizing the health insurance system**	
P-3	*My financial status does not conform with my self-care and thus I modified my self-care autonomously in the absence of my physician's input.*
P-5	*Regrettably, I couldn't even access my refill medications via my health insurance. I believe that the health insurance system needs to be well organized, controlled, and pragmatically implemented.*
P-2	*I would like to have a financial help to pay for the frequently asked lab tests. Otherwise, I can't afford to do it all by myself.*

Supplementary Table S5. Health Care Workers' Propositions to Decrease Treatment Burden and Regimen Fatigue

**Theme-1: Improving self-care and modifying treatment regimens**	
**Subtheme-1: Improving consultation content**	
HCP-1	*Instead of prescribing similar medications all the time, physicians should provide a detailed explanation for patients regarding appropriate medication administration.*
HCP-2	*I think counseling on prescribed medication adherence along with its potential merits outweighs mere prescribing.*
HCP-3	*Both the prescribing physician and dispensing pharmacist should counsel patients regarding drug interactions along with necessary precautions to be followed.*
**Subtheme-2: Providing patient education**	
HCP-4	*Health education on various topics related to DM and HIV/AIDS such as diabetic foot care, HIV transmission modes, and importance of medication adherence etc. should be provided to diabetic and HIV patients at the respective clinics.*
**Subtheme-3: Establishing formalized patient support group system**	
HCP-5	*Creating formalized groups or patient support group systems whereby older and experienced diabetic and HIV patients share their tips and methods to live with DM and/or HIV and its care would be helpful to strengthen patient interactions.*

**Subtheme-4: Availing fixed-dose combination (FDC) medications**	
HCP-6	*Combined medicine preparations should be adequately procured to decrease pill burden (medication-related burden) while enhancing patient compliance.*
HCP-3	*As much as possible, fixed-dose combination (FDC) medications must be available in various government hospitals and Kenema pharmacies at a reasonable price not only to prevent medication-related burden but also a financial related burden.*
**Theme-2: Advancing the administrative services of the clinic and the hospital (TASH)**	
**Subtheme-1: Extending follow-up schedule**	
HCP-6	*If possible, the hospital's OPD pharmacy should be located in near proximity to the health insurance office so as to avoid discomfort related to frequent traveling.*
HCP-7	*To alleviate patients' treatment burden, supply of medications* should be improved, *follow-up schedule should be full-day based, fully functional crucial laboratory tests must be made available in the hospital.*
HCP-8	*Some diabetic patients believed that the consultation time isn't sufficient to undergo a complete checkup and need the follow-up schedule* to be extended.
HCP-9	*The reason behind extended appointment schedule (every 6 month) is not because patients are stable, rather it is due to excess number of patients visiting the clinic. Thus, it is good if patients are scheduled at least every three months unless they are presented with complications associated to the disease.*
**Subtheme-2: Improving laboratory services of the hospital**	
HCP-1	*I heard frequent complaints from patients pertaining to the service provided in the hospital's laboratory. To surmount this, many competent and well qualified lab technicians should be employed. If possible, because the diabetic clinic is one of the heavily burdened clinics in TASH, it is good to establish a separate lab for the clinic where only DM related tests will be performed.*
HCP-1	*Essential medications must be routinely available in the hospital. Patients with health insurance should be warmly welcomed in every private pharmacy* including *Kenema pharmacy and should be supported to access their refill medications.*
HCP 10	*There is shortage of computers in the DM clinic. The number of functional desktop computers should be added to speed-up the ambulatory health care service.*

Theme-3: Promoting the health care system provision	
**Subtheme-1: Establishing a link between health insurance office and Kenema pharmacy**	
HCP-6	*The health insurance office located at office number 55 of the hospital should work hand-in-hand with Kenema pharmacies in town.*
HCP-5	*Prescription errors due to incomplete prescription can be effectively prevented by improving the linkage system of health insurance office with private pharmacies.*
**Subtheme-2: Advancing the I-Care system**	
HCP 2	*The updated I-Care software prescription has no empty space for writing patients' medical diagnosis, and putting prescriber signature. As a result of this, dispensing pharmacists often considered it as incomplete prescription and return patients to respective physician for rectification.*
HCP 4	*Unlike the previous version, prescriptions cannot be copy-pasted in the updated version instead they should be written word by word by the physicians contributing to slow ambulatory care service. Thus, the software should be updated once again addressing these concerns.*
**Subtheme-2: Establishing a well-organized health insurance system**	
HCP-9	*Even if clinically stable patients with health insurance are prescribed 6month refill medication, they are given only a 3month medications due to shortage of supply. Thus, establishing a well-organized health insurance system, sufficiently availing medications in Kenema pharmacies at every corner of the country while at the same time improving patients' economy, and widely establishing pharmacies that provide free or discount service will have uttermost importance to reduce treatment burden.*
**Subtheme-3: Providing financial support**	
HCP-5	*Creating opportunities for patients to obtain medications via sponsorship or health insurance system would be helpful to alleviate the financial burden of patients.*
HCP-10	*Ethiopian Diabetic Association should not interrupt providing free medication to diabetic patients and appreciate medications to be manufactured in local settings.*

I. <u>Interview Guide for Patients (English Version)</u>

1. Could you tell me about your health problems?
   - *What types of health problems are you dealing with right now?*
   - *What do you know about HIV/DM?*
2. What do you to take care of yourself and the disease?
   - *What type of monitoring does you do and how often?*
   - *How do you monitor your symptoms and response?*
3. What kinds of impacts do you experience in the management of the disease?
4. What type of treatment burden did you experience? regimen fatigue?
   - *(Medication related burden, Life style related burden, Financial related burden, Administrative related burden, Social related burden)*
5. What kind of supports did you get from your health care provider to address this problem?
6. What kind of measures you made by yourselves to cope up with this problem?
7. What do you believe are the most important things to change in your care to improve your burden of treatment and reduce your treatment regimen fatigue?
   - *Potential solutions?*
8. Is there anything else that you would like to add about treatment burden and treatment regimen fatigue?

II. <u>Interview Guide for Health Care Workers (English Version)</u>

1. Have you ever encountered a DM/HIV patient facing treatment burden and treatment regimen fatigue? How did you support him/her?
   (i.e., After explanation about treatment burden and treatment regimen fatigue)
2. What do you think are the contributing factors/reasons for the occurrence of treatment burden and regimen fatigue in DM/HIV patients?
3. What do you think are the effects/consequences of excessive treatment burden and regimen fatigue in DM/HIV patients?
4. How might treatment burden and regimen fatigue be decreased in DM/HIV patients? What possible recommendations do you suggest?
5. Of the possible recommendations you have described above, which do you think are easy to implement at low cost in our setup?
